# Co‐Occurrence Patterns of Aquatic Macroinvertebrates in Laurentian Great Lakes Coastal Wetlands

**DOI:** 10.1002/ece3.70622

**Published:** 2024-12-02

**Authors:** Alexandra A. Bozimowski, Brent A. Murry, Donald G. Uzarski

**Affiliations:** ^1^ Institute for Great Lakes Research Central Michigan University Mount Pleasant Michigan USA; ^2^ Division of Forestry and Natural Resources, Davis College West Virginia University Morgantown West Virginia USA

**Keywords:** community assembly, competition, null models

## Abstract

In niche‐based community assembly theory, it is presumed that communities in habitats with high natural disturbance regimes are less likely to be structured by competitive mechanisms. Laurentian Great Lakes (hereafter Great Lakes) coastal wetlands can experience drastic diel fluctuations in dissolved oxygen levels, severe wave action, ice scour, and near complete freezing during the winter such that conditions are inhospitable for most organisms. The high natural disturbance levels are thought to cause high interannual turnover for aquatic macroinvertebrate communities and support the hypothesis that these communities are less likely to experience less competitive interactions and negative co‐occurrence structure. We hypothesize that non‐random co‐occurrence patterns will be rare in Great Lake coastal wetlands and non‐competitive processes (e.g., through shared or differential microhabitat affinities, pollution tolerances, or biotic homogenization) will be more common than competitively driven negative co‐occurrence patterns. Null model analysis was performed on 134 macroinvertebrate communities sampled from across the Great Lakes basin from 2000 to 2013. To disentangle the effects of alternative structuring mechanisms (i.e., shared/differential habitat affinities, shared/differential pollution tolerance, and biological homogenization/competitive exclusion), communities were parsed based on the year sampled, the vegetation type from which community samples were collected, and lastly species' functional feeding group assignment or taxonomic group. As expected, very few communities were non‐randomly structured; however, all of those that were non‐random exhibited showed more negative co‐occurrences than by chance. Upon further investigation, these communities consisted of species that are known to overwinter in wetlands, and therefore, avoid having to recolonize after each spring thaw. With expected changes in habitat conditions due to climate change, we propose that null model analyses can be used as an early warning system for community change.

## Graphical abstract

We investigated community structure and species co‐occurrence patterns in Great Lakes coastal wetlands benthic macroinvertebrates using null models. We developed a framework and approach to address recent controversy over the use of presence‐absence matrices and null models to infer ecological interactions from non‐random co‐occurrence patterns using community data collected over thirteen years from 172 sites across the Great Lakes basin. Very few communities showed non‐random co‐occurrence patterns; however, the majority of non‐random situations contained invertebrate taxa known to over‐winter. We discuss how these taxa may be driving the negative co‐occurrence patterns we detected and suggest that results from presence absence matrices and null model analysis could be used as an early warning system in the face of climate change.

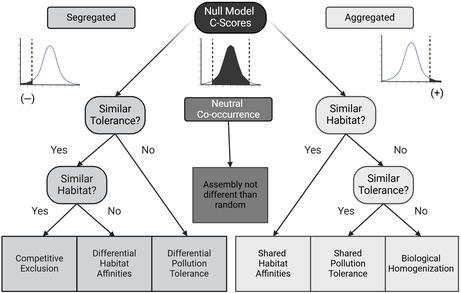

## 1 Introduction

In niche‐based community assembly theory, competition is the dominant structuring mechanism (Weiher et al. 2011). Competition drives community structure in most habitats where resources are limited (Hairston, Smith, and Slobodkin 1960) and may lead to patterns of negative co‐occurrence (Diamond 1975). However, if an assemblage or community is not at carrying capacity, such as in high‐disturbance systems, the structuring capacity of competitive interactions is presumed to be less (Connell 1978). Alternatively, whether at carrying capacity or not, there is growing evidence of positive relationships among species facilitated by introduced and invasive species (Albertson et al. 2021; Ricciardi 2001) or by shared habitat affinities (Davis et al. 2018; Gotelli, Buckley, and Wiens 1997).

Community assembly and co‐occurrence patterns have been used as diagnostic tests to understand competition‐driven structure, where co‐occurrences (or lack thereof) are attributed to the effects of species interactions. Co‐occurrence patterns are commonly assessed using null model analysis of checkerboard patterns (Connor and Simberloff 1983; Gotelli 2000). Diamond (1975) first pointed out checkerboard distributions among birds of the Bismarck Islands, where species of the same genus rarely co‐occurred leading to negative co‐occurrence patterns, or the checkerboard distribution. The checkerboard is derived from opposing presence–absences among paired species (i.e., where Species X is present Species Y is not, and vice versa). These observations led him to suggest that checkerboard distributions were an indicator of competition. Later research expanded upon these core ideas to identify alternative means of non‐random, negative, or positive pairwise species associations including habitat and pollution affinities (Davis et al. 2018; Gotelli, Buckley, and Wiens 1997). Co‐occurrence patterns are primarily tested through null models (Gotelli 2000) which compare the observed number of checkerboards (or other similar metrics) among a suite of taxa and sites to a randomized distribution of the same matrix. Significant non‐random co‐occurrence patterns (often called community structure) occur when the observed number of checkerboards is found in the top (positive) or bottom (negative) 2.5% of the random distribution (Gotelli and Entsminger 2006).

Gotelli and McCabe (2002) performed a meta‐analysis of non‐social invertebrate populations using null models and generally found a co‐occurrence structure that did not differ from that expected by chance; however, the effects of site variation may have diluted any significant interactions (Blanchet, Cazelles, and Gravel 2020). Consideration of alternate mechanisms driving community assembly (e.g., environmental, temporal, or indirect species interactions) should also be explored prior to using common community assembly analyses to avoid misinterpretation of results toward competitive interactions (Blanchet, Cazelles, and Gravel 2020). For example, habitat preferences and geographic origin (e.g., recent introductions and invasions) can readily obscure competition‐based predictions.

Within coastal wetlands, aquatic macroinvertebrates comprise most macro‐organisms (Chase 2007) and are present within benthic, planktonic, and epiphytic communities (Krieger 1992). Macroinvertebrate community composition is strongly correlated with vegetation type, as well as wind exposure and subsequent wave action (Krieger 1992; Burton, Stricker, and Uzarski 2002; Burton, Uzarski, and Genet 2004). Gathman (2000) suggested mechanisms such as predation and competition would shift relative to wave exposure within coastal wetland invertebrate communities. Similarly, most macroinvertebrates found in wetlands do not complete their entire life cycle in the wetlands, therefore, it may be unlikely that competition drives community structure toward negative co‐occurrence, although that speculation is countered by observations that stream invertebrates may occasionally experience competitive interactions, especially at local scales (Holomuzki, Feminella, and Power 2010). Seasonal shifts in wetland habitat conditions are profound where productivity and biological diversity are high during the summer and severely constrained in the winter. Ice frequently forms throughout the water column down to the substrate. These harsh conditions are hypothesized to be the main driver of high site‐level turnover (Langer et al. 2016; Stewart and Schriever 2022).

**FIGURE 1:**
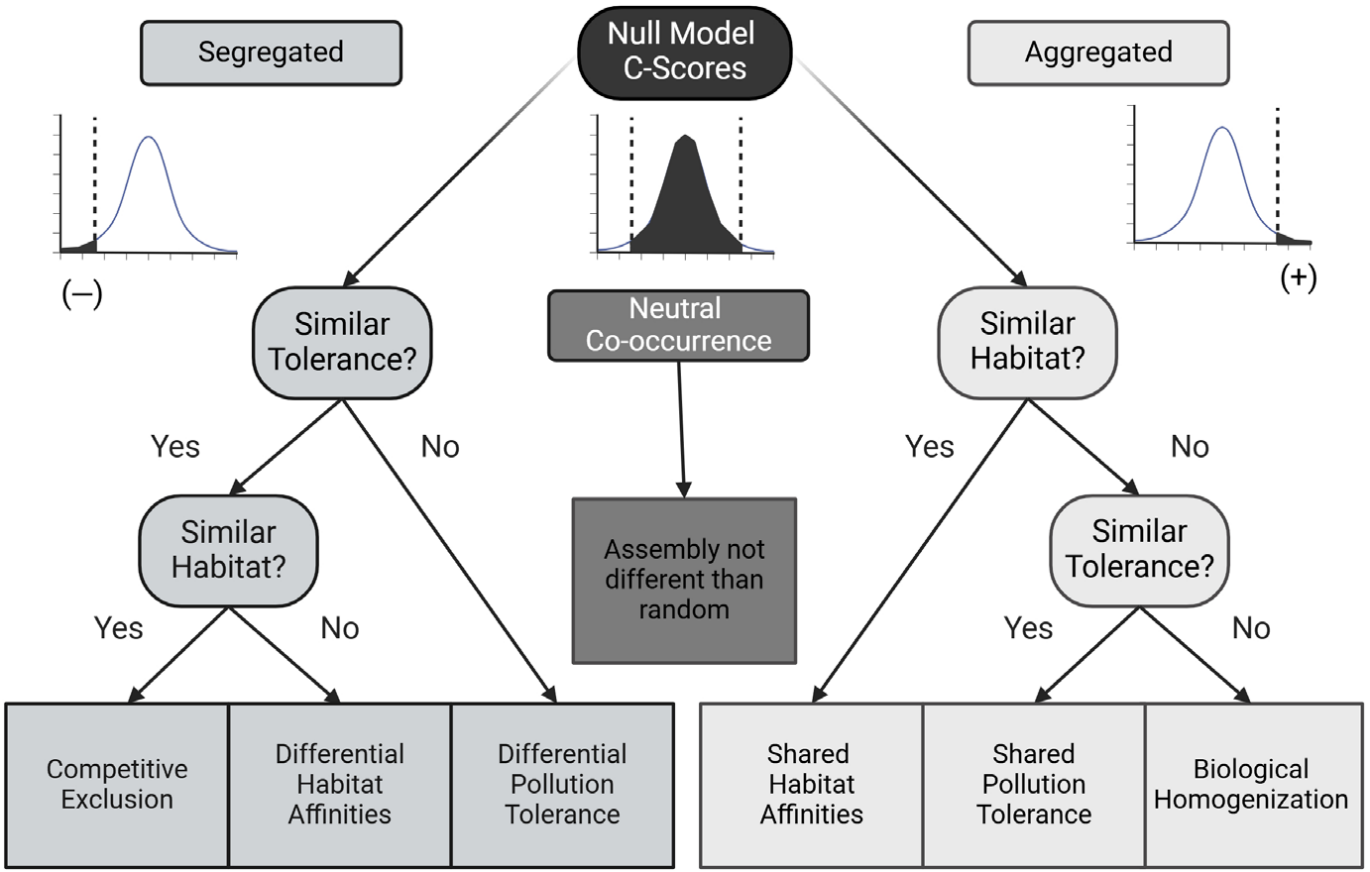
Consistent with Table 1, null model analysis of trophic or taxonomic groupings within appropriate environmental strata (year, ecoregion, and vegetation zone) resulted in one of three outcomes, non‐random negative (left side), non‐random positive (right side), or assemblages that do not differ from random (center of figure). These represent segregation, aggregation, or neutral co‐occurrence, respectively. Subsequently, this decision matrix was used to assess the C‐score of each interactive pair designed to systematically isolate all alternative hypotheses for the observed relationship.

Global change, including rampant habitat alterations, more variable weather patterns, and intensifying fire, snow, and storm regimes are changing the historic stability of systems (Hautier et al. 2015) and potentially altering historic patterns of community assembly. Great Lake coastal wetlands offer a unique and important model system for evaluating community assembly (Weiher and Keddy 2001), and specifically co‐occurrence patterns in highly dynamic and hostile environments that may be insightful in our rapidly changing world. Given the high interannual turnover, high natural disturbance regimes (e.g., wave action and water chemistry), and frequently high anthropogenic disturbances, we should realistically expect co‐occurrence patterns to predominately occur by chance among functionally and taxonomically similar species. We hypothesize that non‐random co‐occurrence is rare in Great Lakes coastal wetlands and due to special circumstances more likely driven by microhabitat affinities, pollution tolerance, or biotic homogenization than by competition. Clearly, each of these operates simultaneously and synergistically—however, we chose to isolate them to determine their relative influence on community structure. Here, we evaluate the co‐occurrence patterns of macroinvertebrates within Laurentian Great Lakes coastal wetlands and offer potential alternative mechanisms for non‐randomly structured communities (Table 1, Figure 1).

**TABLE 1:** Predictive framework to utilize presence–absence data from ongoing monitoring efforts to provide a different lens into wetland macroinvertebrate community structure.

Null model analysis outcome	Hypothesized mechanism	Supporting evidence	Hypothesis test
Positive co‐occurrence (aggregated)	Biological homogenization	High abundance of non‐native and/or generalist species	N/A^a^, Process of elimination: positive co‐occurrence and lack of shared habitat affinities
	Shared habitat affinities—similar niche	Relatively benign or low disturbance habitat, higher species richness, diversity of adaptations, positive co‐occurrence restricted to fewer species	N/A^a^, PERMANOVA test of environmental variables among sites involved in negative co‐occurrence pattern
	Shared habitat affinities—similar pollution tolerance	Highly disturbed habitat, low species richness, and similar disturbance‐related adaptations, positive co‐occurrence among most species in habitat	N/A^a^, compare pollution tolerance scores^b^ among taxa involved with checkerboard co‐occurrence pattern
Random (neutral)	Unstructured community assembly, not different than random, likely not up to carrying capacity	Wetlands are one phase of the life cycle of most macroinvertebrates, winter inhospitable conditions, spring recolonization, and high species turnover	Self‐evident if null model analysis is insignificant
Negative co‐occurrence (segregated)	Competitive exclusion	Overlap in species niche space	Process of elimination: negative co‐occurrence and lack of differential tolerance and differential habitat affinities
	Differential tolerance to pollutants	Sites containing or lacking pollution‐sensitive species	Compare pollution tolerance scores^a^ among taxa involved with checkerboard co‐occurrence pattern
	Differential habitat affinities	Differences exist in microhabitat conditions among sites that favor different species, e.g., water chemistry, vegetation density	PERMANOVA test of environmental variables among sites involved in negative co‐occurrence pattern

*Note:* Although recent arguments (Blanchet, Cazelles, and Gravel 2020) suggest that co‐occurrence observations do not provide evidence of ecological interactions, here we argue that they still provide insights into community change that can not only identify change but also provide insights into potential causes that can guide further research and conservation actions. Recognizing that these drivers operate simultaneously, we isolate and partition them to test hypothesized drivers individually (Figure 2). This table assumes that habitat and species groups are controlled or categorized to focus on meaningful co‐occurrence patterns (i.e., avoid spurious associations driven by different habitats). For example, in this study, we constrained sites to within accepted ecoregions and specific wetland types and species comparisons within functional groups.

^a^ N/A: For this study, this is not applicable as we observed no positive co‐occurrence among taxa.

^b^ Pollution tolerance scores were taken from Hilsenhoff (1987), Hilsenhoff (1988), and Merritt and Cummins (2019).

## 2 Methods

### 2.1 Data Collection

**FIGURE 2:**
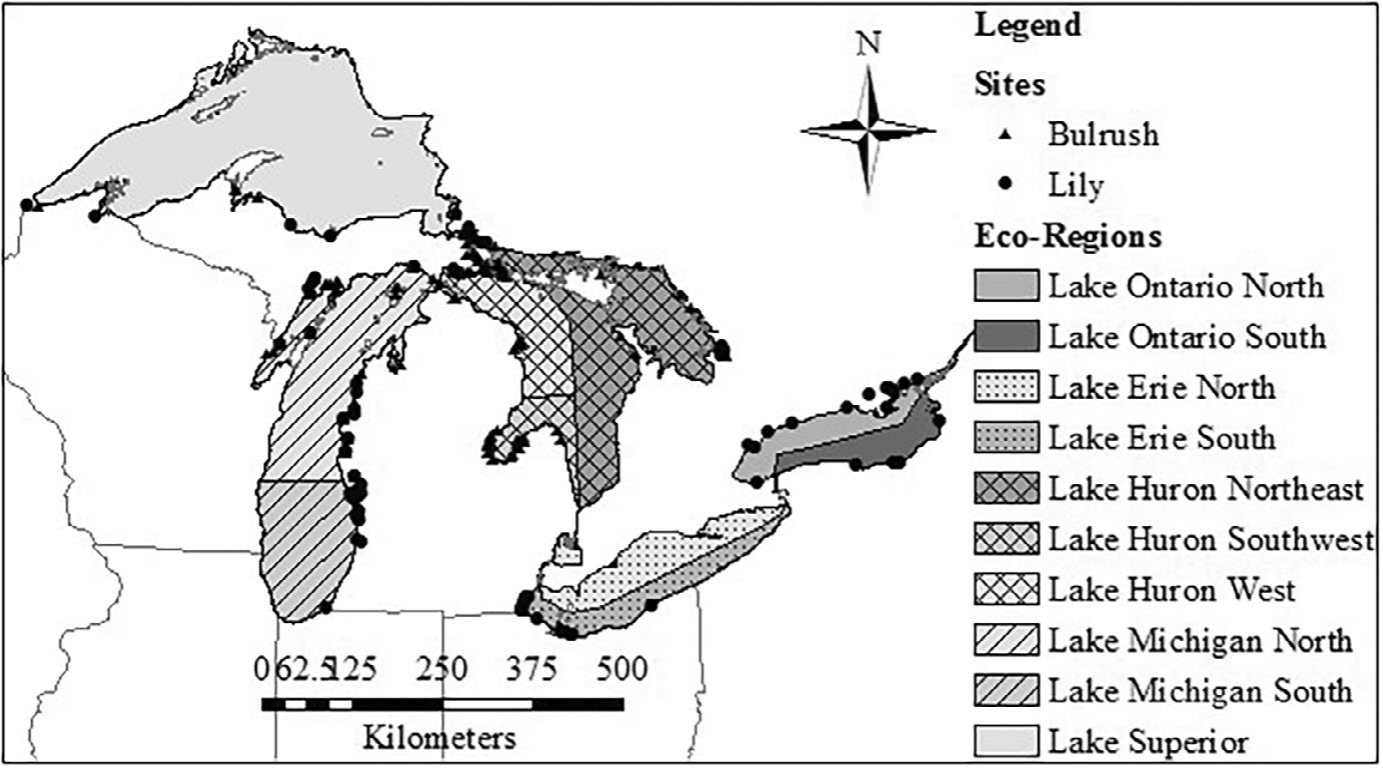
Map showing all historical bulrush and lily sites from 2000, 2002, 2004–2005, 2008, and 2010–2013. Sites spanned nine eco‐regions, although all 10 regions are represented.

Macroinvertebrate abundance data and associated site physical/chemical data were collected by several Great Lakes coastal wetland monitoring endeavors (Burton et al. 1999; Cooper et al. 2006; Cooper, Uzarski, and Burton 2007; Uzarski, Burton, and Genet 2004). Macroinvertebrates were sampled from dominant plant zones (e.g., Submerged Aquatic Vegetation, Lily, Bulrush) at wetland sites throughout the Great Lakes basin between June and early September dependent upon the latitude of the given location. Samples were collected by sweeping D‐frame dipnets with 500‐μm mesh bags throughout the entire water column and against vegetation and benthos. Net contents were emptied into gridded trays and technicians picked specimens from individual grid‐squares until the square was devoid of individuals. Organisms were preserved in labeled vials of ethanol and identified in the laboratory. For this work, macroinvertebrate data from 241 wetland sites over 9 years across the Great Lakes Basin (Figure 2) were used to build community matrices for null model analysis.

Environmental data associated with macroinvertebrate samples included dissolved oxygen (mg/L), water temperature (C), pH, specific conductance (μS/cm), total alkalinity, turbidity (NTU), soluble reactive phosphorus (μg/L), and ammonia/ammonium (μg/L). Environmental data for wetlands sampled between 2010 and 2013 were collected via the Coastal Wetland Monitoring Program (CWMP), a Great Lakes Basin‐wide monitoring effort (Uzarski et al. 2017). Additional parameters collected through CWMP included chlorophyll‐a (mg/L), total phosphorus (mg/L), total nitrogen (mg/L), and nitrate/nitrite (mg/L). A “SumRank” metric representing anthropogenic disturbance for the given vegetation zone was calculated by Uzarski et al. (2005) and refined by Cooper et al. (2018) and included in the environmental dataset. The “SumRank” metric is a composite index of physical–chemical variables and four land‐cover classes that were assembled by principle component analysis and summing across rank‐transformed variables for each of the major vegetation zones. This index represented a disturbance regime where higher SumRank scores corresponded to higher water quality and lower development of adjacent lands (Cooper et al. 2018). We similarly used these data as a measure of disturbance for comparison across sites.

### 2.2 Biological Units: Functional Feeding Groups and Taxonomic Families

We categorized macroinvertebrate communities by functional feeding group and taxonomic group (family; Table 1). Species within the same functional feeding group share similar carbon sources. If a community or assemblage were at carrying capacity, negative co‐occurrence patterns would be more likely to be seen among taxa within the same functional feeding group. Similarly, invertebrate species within the same taxonomic group tend to be within the same functional feeding group and/or have a shared ancestral predisposition that is more likely to result in co‐occurrence interactions. Taxa in the same family also tend to be found within similar geographic areas. Therefore, we further categorized invertebrate community data by functional feeding group assignment and taxonomic family (for a given year, eco‐region, and wetland type). Functional feeding groups included predator, shredder, collector, and grazer.

### 2.3 Null Model Analysis

We used null model analysis to evaluate community assembly patterns, specifically non‐random species co‐occurrence patterns in Great Lakes coastal wetlands. Specifically, we used EcoSim Professional (Gotelli and Entsminger 2006) for null model analysis and compared 5000 iterations of randomized versions of each observed community matrix to the original observed matrix. We chose Stone and Roberts' (1990) C‐score as our co‐occurrence index. The C‐score calculates the average number of checkerboard distributions for an individual observed matrix (i.e., positive or negative presence–absence patterns among all possible species pairwise comparisons). The randomized iterations of the observed matrix (to which the observed matrix is compared) can be manipulated in terms of row and column totals. We chose to fix row‐column values such that the number of taxa found within a given site in any randomized matrix is fixed for the site and the number of times a taxon occurs is fixed within the community. These constraints were chosen because they are documented to yield low Type I errors and are considered the most robust and conservative option (Gotelli 2000). Since each taxon occurs exactly as many times in the randomized matrices as it does in the observed matrix and each column (or site) contains the same number of taxa as in the observed matrix, we maintain differences that were observed among taxa and sites in the null model. This allowed us to measure any additional patterns beyond those differences seen in the observed matrix (Gotelli 2000). The Sequential Swap randomization algorithm was used whereby two‐by‐two submatrices were randomly exchanged within the matrix while still maintaining row and column totals. This method has been shown to yield low Type I errors when used with the C‐score index and the fixed–fixed constraints upon randomized matrices (Gotelli and McCabe 2002).

**FIGURE 3:**
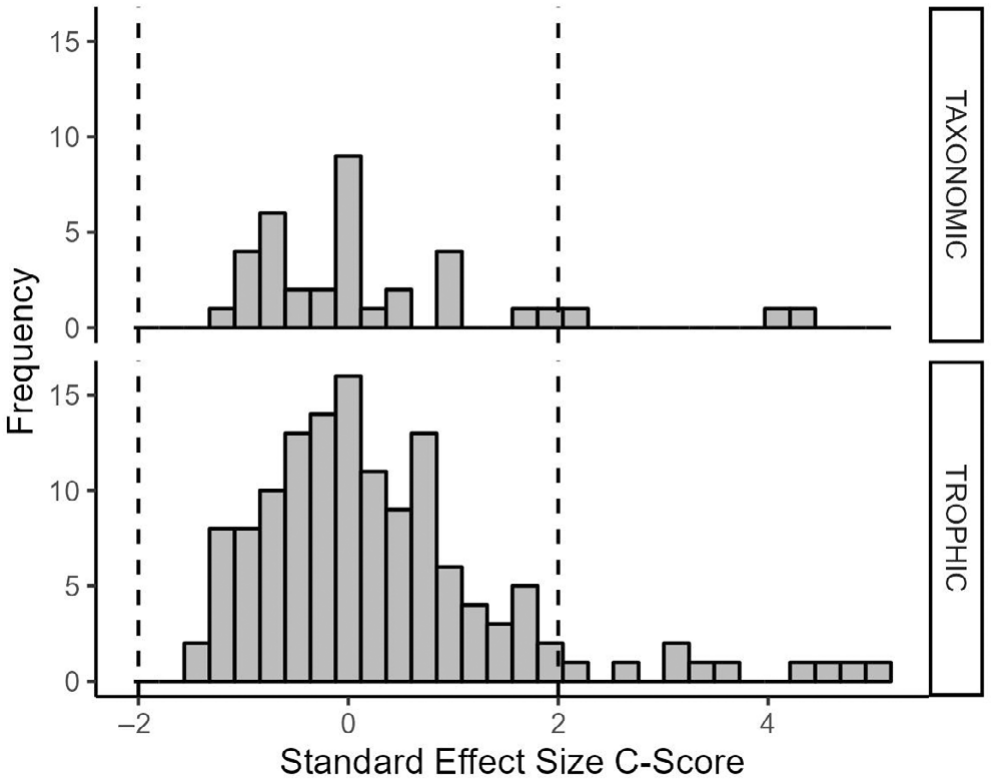
Frequency distributions for standardized effect sizes (SES) for the C‐score index for matrices organized by taxonomic classification (Family) and trophic group (functional feeding group). The null hypothesis is that the average effect size is zero and that 95% of observations lie between −2 and +2 (Horner‐Devine et al. 2007). The SES was not significantly different from the null for the taxonomic group (mean = 0.2496, *H* = 5.5523, *p* = 0.851), but was significantly greater than expected for the trophic group (mean = 0.3210, *H* = 9.8867, *p* = 0.020). Vertical dotted lines represent the outer boundaries of the 95% confidence interval given the null hypothesis that the average SES is equal to zero.

To compare the degree of co‐occurrence, we calculated the standardized effect size (SES) for every observed community matrix for both trophic and taxonomic biological units (Gotelli and McCabe 2002; Horner‐Devine et al. 2007). SES is a statistical measure of deviation from random co‐occurrence and was calculated for each observed matrix within EcoSim Professional (Gotelli and Entsminger 2006). In short, SES is calculated for each community matrix as the number of standard deviations of the observed co‐occurrence matrix index score (in our case, the observed C‐score) above or below the mean co‐occurrence matrix index score for all simulated community matrices. We then examined how variation in SES related to both trophic and taxonomic classifications. Since SES distributions were not normally distributed (Figure 3), nonparametric Kruskal–Wallis tests were performed to examine the effects of trophic and taxonomic groupings on SES (Horner‐Devine et al. 2007). We then ran a pairwise Wilcoxon rank‐sum test (aka Mann–Whitney *U*‐test) to determine whether SES differs with respect to functional feeding group or taxonomic family.

Matrix size and statistical power of the analysis are positively correlated (Gotelli and McCabe 2002); however, it has been shown that extremely large matrices (greater than 100 rows by 100 columns) yield significant differences from null models whether or not the dataset is truly randomly structured (Fayle and Manica 2010). On the other hand, it is more likely with very small matrices that Type II errors arise (Gotelli and Ulrich 2012), but most null model literature agrees that it is best to control for Type I errors. Fayle and Manica show that even type II errors for this size of matrices are quite low (< 0.11). No matrix analyzed had greater than 66 rows (taxa) or columns (sites) and were considered small to intermediate in size (Fayle and Manica 2010; Gotelli and Ulrich 2012); however, we found that the probability of sites being inherently degenerate (i.e., having multiple instances of row and column totals equal to zero) and thus uncooperative with the specified co‐occurrence index, row‐ and column‐constraints, and randomization algorithm increased when matrices were smaller than five rows by four columns. Therefore, to be considered for further analysis, observed matrices were required to have at least five taxa (rows) and four sites (columns).

### 2.4 Geographic and Habitat Units

To detangle potential drivers of non‐random co‐occurrence (Table 1), we categorized macroinvertebrate communities by geographic region and dominant vegetation zone/habitat. Site variability may mask significant interactions within a given community. Due to the harsh winters experienced by Great Lakes coastal wetlands, interannual variability is high (Langer et al. 2016; Stewart and Schriever 2022). Therefore, community data were initially separated by year. Furthermore, the Laurentian Great Lakes basin could be separated based on distinct physical–chemical properties influenced by the underlying bedrock into “eco‐regions” (Uzarski et al. 2005). Some taxa may also have such different distributions that if analyzed within the same co‐occurrence matrix, they will inflate negative co‐occurrence structure (Gilpin and Diamond 1982). As such, co‐occurrence matrices were built with consideration for the eco‐region within which a given wetland site was located. Eco‐regions included Lake Superior (LS), Lake Michigan‐North (LMN), Lake Michigan‐South (LMS), Lake Huron‐Northeast (LHNE), Lake Huron‐West (LHW), Lake Huron‐Southwest (LHSW), Lake Erie‐South (LES), Lake Ontario‐North (LON), and Lake Ontario‐South (LOS) (Figure 2).

To mitigate habitat‐related co‐occurrence patterns, community data collected from bulrush (*Schoenoplectus* spp.) and lily (*Nymphaea* or *Nuphar* spp.) vegetation zones were analyzed separately. Bulrush and lily‐dominated wetland sites tend to have disparate natural disturbance regimes. Wetlands with dominant bulrush zones tended to be lacustrine coastal fringing wetlands that experienced high wave energy, had courser substrates (e.g., sand, gravel, and cobble), low organic composition, cooler water temperatures, and higher dissolved oxygen levels (Albert and Minc 2004; Albert et al. 2005). In contrast, lily‐dominated wetlands were more commonly found in drowned river mouth systems and protected embayments. They tended to experience lower wave action, had fine and highly organic substrates (e.g., silt and mud), warmer temperatures, and often had very low dissolved oxygen levels (Albert and Minc 2004, Albert et al. 2005). In summary, data were initially partitioned into discrete nested units based on year, ecoregion, and wetland type as the basis for evaluating potential mechanisms for assembly structure in Great Lakes coastal wetlands.

### 2.5 Investigating Instances of Non‐random Community Structure

The categorization of the invertebrate community dataset by geographic and habitat units and then into functional feeding groups and taxonomic family groups acknowledged our original hypotheses as well as considerations brought forth by Blanchet, Cazelles, and Gravel (2020). Blanchet et al. presented arguments on how presence–absence data are likely to be misinterpreted to incorrectly assume species interactions. Separating our community data by distinct geographic eco‐regions and habitat types removed influences of well‐documented species biotic preferences that would appear as strong co‐occurrence signals in null model analyses. However, we acknowledge that unknown or micro‐abiotic influences could still potentially result in non‐random co‐occurrences. Categorizing community data by ecoregion and habitat type (i.e., macrohabitat) and then by taxonomic or functional feeding groups (in separate analyses) focused the analyses on potential competitive interactions between species likely sharing similar carbon sources while reducing the influence of indirect species interactions (Figure 1). For those communities where the C‐score was significantly different from random, we investigated EcoSim Professional output of the number of checkerboard distributions per taxon (used to determine average distribution per matrix). Often, a singular taxon would arise within the observed presence–absence matrix with considerably more checkerboard distributions than other taxa within the matrix, presumably caused by the given taxon being present at only one site where all or most other taxa were not present and subsequently being absent from all other sites where other taxa were all or mostly present (see Data S1).

We used the decision tree illustrated in Figure 1. to isolate alternative mechanisms to observe non‐random co‐occurrence. In cases of non‐random positive co‐occurrence, the right side of the tree is followed by testing for shared/differential microhabitat affinities (macrohabitats were controlled for by the study design) and then for similar or opposing pollution tolerances. Alternatively, when the non‐random co‐occurrences were negative, we first test for differences (or similarities) in pollution tolerance/sensitivity scores and then evaluate shared/differing microhabitat affinities.

Pollution tolerance/sensitivity scores were assigned to all taxa within non‐random community matrices based on standard texts (Hilsenhoff 1987, Hilsenhoff 1988, Merritt and Cummins 2019). If taxa with differing pollution tolerance values also showed increased checkerboard distribution (thus, driving negative co‐occurrence), segregation based solely on competitive exclusion was refuted. However, if taxa showed similar tolerance values, competitive exclusion may be driving community assemblies (Figure 1).

Likewise, for each instance where non‐random community structure was detected, permutational multivariate analysis of variance (PERMANOVA) was used to test for significant differences in site physical/chemical attributes. There were several instances of a taxon occurring at only one or a handful of sites within a matrix that also showed increased checkerboard distributions (i.e., negative co‐occurrence) with one or several other taxa. Specifically, within the community matrix for Year:2000, Ecoregion: LMN, Vegetation: Bulrush, FFG: Collectors, site 1281; within the community matrix for Year: 2011, Ecoregion: LHW, Vegetation: Lily, FFG: Collectors, site 816; within the community matrix for Year: 2011, Ecoregion: LS, Vegetation: Bulrush, FFG: Predators, sites 969 and 5210; within the community matrix for Year: 2012, Ecoregion: LHNE, Vegetation: Bulrush, FFG: Predators, site 5900; within the community matrix for Year: 2013, Ecoregion: LHW, Vegetation: Bulrush, FFG: Grazers, site 777; and, within the community matrix for Year: 2012, Ecoregion: LHW, Vegetation: Bulrush, FFG: Collectors, sites 496, 636, and 721. All community matrices are available via data release referenced within the Data Availability section. Taxon presence was used as the grouping variable to determine whether a given site showed significantly different physical/chemical attributes. If the site was determined to be significantly different than those where the taxon did not occur, negative co‐occurrence due to competitive exclusion was refuted due to the higher likelihood of differential habitat preferences of the species pairs. Environmental data for all sites within a given matrix were first reduced to three principal components using the *prcomp* function in R (Holland 2008; Table 2). Environmental PCs were then partitioned into groups based on the site(s) at which taxa with high species checkerboard distributions were present for significance testing via PERMANOVA.

**TABLE 2:** Principle component (PC) loadings, standard deviation, and proportion of variance explained.

Community matrix	Variable	PC1	PC2	PC3
Bulrush, 2000, LMN, collectors	Dissolved oxygen	0.3195	0.4256	0.5907
	Temperature	0.1582	−0.5141	0.6113
	pH	−0.5620	−0.0111	0.3539
	Specific conductance	−0.4489	0.3408	0.3535
	Soluble reactive phosphorus	0.5783	−0.0075	0.1448
	Ammonia/ammonium	−0.1456	−0.6620	0.0794
	Standard deviation	1.6518	1.4204	1.0053
	Proportion of variance	0.4547	0.3363	0.1684
Lily, 2011, LHW, collectors	Dissolved oxygen	−0.3072	−0.2876	−0.1755
	Temperature	0.2534	−0.4055	−0.0361
	pH	0.0657	−0.4920	−0.3540
	Specific conductance	−0.3564	0.1436	−0.0779
	Total alkalinity	−0.3338	0.2296	0.1130
	Chlorophyll‐α	0.3614	−0.0415	0.1715
	Total phosphorus	0.2710	0.3669	−0.1440
	Total nitrogen	0.3275	0.1537	−0.3095
	Ammonia/ammonium	−0.3572	0.0725	−0.1911
	Nitrate/nitrite	−0.1204	−0.1235	0.7521
	SumRank	−0.3442	0.0899	−0.2733
	Fetch	0.1638	0.4989	−0.0304
	Standard deviation	2.6977	1.7967	1.2225
	Proportion of variance	0.6065	0.2690	0.1245
Bulrush, 2011, LS, predators	Dissolved oxygen	0.3494	−0.1147	−0.3916
	Temperature	−0.2624	0.4034	0.0745
	pH	−0.0009	0.4218	−0.4126
	Specific conductance	−0.3322	−0.2610	−0.2775
	Total alkalinity	−0.2845	−0.3434	0.2475
	Chlorophyll‐α	−0.3622	−0.2522	0.1434
	Soluble reactive phosphorus	−0.1731	−0.1383	−0.4394
	Total nitrogen	−0.3291	−0.0508	−0.4372
	Ammonia/ammonium	0.2959	−0.3293	−0.1382
	Nitrate/nitrite	0.3761	−0.1551	−0.2125
	SumRank	0.3408	0.0394	0.2475
	Fetch	−0.0565	0.4908	−0.0478
	Standard deviation	2.3182	1.9469	1.3834
	Proportion of variance	0.4479	0.3159	0.1595
Bulrush, 2012, LHNE, predators	Dissolved oxygen	−0.3350	−0.2882	−0.0715
	Temperature	0.1309	−0.2070	−0.6454
	pH	−0.3450	−0.2284	−0.1014
	Specific conductance	−0.3716	−0.0986	−0.1338
	Total alkalinity	−0.0695	0.4491	−0.4312
	Chlorophyll‐α	0.3450	−0.1246	−0.2462
	Total phosphorus	0.3770	0.0347	0.0770
	Total nitrogen	0.3761	−0.0427	−0.0949
	Ammonia/ammonium	−0.1218	−0.4438	0.1770
	Nitrate/nitrite	−0.1755	0.5050	−0.1903
	SumRank	−0.3381	−0.0211	−0.3040
	Fetch	−0.2150	0.3736	0.3587
	Standard deviation	2.6077	1.6355	1.3560
	Proportion of variance	0.5667	0.2229	0.1532
Bulrush, 2013, LHW, grazers	Dissolved oxygen	0.3183	0.2375	0.2641
	Temperature	0.1365	−0.1825	−0.6128
	pH	0.3209	0.3412	0.0088
	Specific conductance	−0.3070	0.3223	0.2452
	Total alkalinity	−0.1984	0.3958	0.2766
	Total phosphorus	−0.1287	−0.3630	0.3543
	Soluble reactive phosphorus	−0.2099	−0.1824	0.2572
	Total nitrogen	0.1510	−0.3427	0.4127
	Ammonia/ammonium	−0.4372	−0.0832	−0.1206
	Nitrate/nitrite	−0.0450	−0.4786	0.0752
	SumRank	0.4168	−0.0890	0.1301
	Fetch	−0.4395	0.0838	−0.1379
	Standard deviation	2.1975	2.0521	1.3362
	Proportion of variance	0.4024	0.3509	0.1488
Bulrush, 2012, LHW, collectors	Dissolved oxygen	0.3183	0.4786	−0.1314
	Temperature	0.4180	0.2520	−0.2668
	pH	0.4470	0.1693	0.2212
	Total alkalinity	−0.3849	0.2700	−0.3971
	Total phosphorus	0.2548	−0.2744	−0.4179
	Soluble reactive phosphorus	−0.4065	0.0974	−0.4484
	Total nitrogen	−0.0692	0.5814	−0.1304
	Ammonia/ammonium	−0.3610	0.1009	0.3932
	Nitrate/nitrite	−0.1040	0.4157	0.3971
	Standard deviation	1.8460	1.6445	1.3038
	Proportion of variance	0.3786	0.3005	0.1889

*Note:* PCs 1 through 3 were then partitioned into groupings based upon sites within the given community matrix showing non‐random co‐occurrence patterns where one or several taxa showing high checkerboard distributions (i.e., negative co‐occurrence) were present.

Competitive exclusion was only suggested as the driving mechanism between negative co‐occurrence patterns when there were also no differences in pollution tolerance among the involved taxa and no difference in microhabitats among involved sites.

## 3 Results

### 3.1 Null Model Analysis

The structure of macroinvertebrate communities in the majority of Great Lakes coastal wetlands was not differentiated by assembly through chance, that is, the null model was seldom rejected across nearly all functional feeding groups and taxonomic groups. In total, 134 macroinvertebrate communities were analyzed, of which only nine showed non‐random co‐occurrence structures (6.7%). All instances of non‐random community structure were negative. It should be noted here that each individual matrix, while unique and independent of the others can be affected by its own internal type I and type II errors (Fayle and Manica 2010; Gotelli and Ulrich 2012), thus there is a chance that some portion of the nine matrices showing non‐random structure may be by chance. All functional feeding groups were represented among the community matrices that exhibited significantly negative taxon co‐occurrence patterns (Table 3); however, the majority were from the Collector (4 of 9) and Predator (3 of 9) functional feeding groups. Of 36 community matrices categorized by taxonomic group (Family), only two showed a non‐random (negative) co‐occurrence structure (Tables 4 and 5). Eleven families were represented when macroinvertebrate data were categorized taxonomically. Taxa within Chironomidae (1 of 2) and Planorbidae (1 of 2) were negatively structured.

**TABLE 3:** Null model analysis results based upon the C‐score index for macroinvertebrate community matrices categorized by trophic (functional feeding) group.

Year	Ecoregion	Vegetation	Sites		Collectors	Grazers	Predators	Shredders
2000	LHW	Bulrush	4	No. Taxa	16	8	16	10
				C‐score	0.4500	0.5357	0.6500	0.5111
				*p* (obs ≤ expected)	0.7338	0.8332	0.3346	0.9020
				*p* (obs ≥ expected)	0.5622	0.6424	0.8138	0.2396
2000	LMN	Bulrush	5	No. Taxa	**13**	6	24	10
				C‐score	**1.0385**	1.0000	1.1413	0.6444
				*p* (obs ≤ expected)	**0.9922**	0.9306	0.9446	0.8436
				*p* (obs ≥ expected)	**0.0104**	0.3618	0.0622	0.2706
2000	LMN	Lily	6	No. Taxa	13	—	18	10
				C‐score	1.2308		0.7778	0.5333
				*p* (obs ≤ expected)	0.3692		0.2452	0.7974
				*p* (obs ≥ expected)	0.7378		0.8332	0.2654
2002	LHSW	Bulrush	6	No. Taxa	14	6	31	6
				C‐score	0.6264	0.2667	0.9871	0.6000
				*p* (obs ≤ expected)	0.3178	1.0000	0.4312	0.2056
				*p* (obs ≥ expected)	0.8322	1.0000	0.5893	1.0000
2002	LHW	Bulrush	11	No. Taxa	19	14	**37**	17
				C‐score	1.9942	1.8352	**2.7072**	1.9632
				*p* (obs ≤ expected)	0.1352	0.5218	**0.9996**	0.4578
				*p* (obs ≥ expected)	0.8882	0.5324	**0.0004**	0.5880
2002	LMN	Bulrush	10	No. Taxa	22	12	35	**15**
				C‐score	2.6494	2.4394	2.0891	**2.0571**
				*p* (obs ≤ expected)	0.8726	0.7836	0.7448	**0.9880**
				*p* (obs ≥ expected)	0.1392	0.2550	0.2712	**0.0146**
2002	LMN	Lily	6	No. Taxa	10	9	29	12
				C‐score	0.7333	0.9167	1.0640	0.7273
				*p* (obs ≤ expected)	0.4586	0.2160	0.8022	0.3416
				*p* (obs ≥ expected)	0.8040	0.8920	0.2154	0.7702
2002	LMS	Lily	5	No. Taxa	9	6	25	10
				C‐score	0.3611	0.8667	0.9333	0.7556
				*p* (obs ≤ expected)	0.9312	0.6958	0.1600	0.2174
				*p* (obs ≥ expected)	0.4850	0.8092	0.9388	0.9466
2004	LHSW	Bulrush	4	No. Taxa	13	—	24	10
				C‐score	0.6282		0.5725	0.6667
				*p* (obs ≤ expected)	0.4456		0.8242	0.6434
				*p* (obs ≥ expected)	0.8444		0.2156	0.6228
2004	LMS	Lily	4	No. Taxa	**10**	—	19	8
				C‐score	**0.9778**		0.8538	0.3929
				*p* (obs ≤ expected)	**0.9960**		0.8334	0.3748
				*p* (obs ≥ expected)	**0.0102**		0.2488	1.0000
2005	LHSW	Bulrush	4	No. Taxa	11	6	15	9
				C‐score	0.3273	0.1333	0.3619	0.4444
				*p* (obs ≤ expected)	0.7372	1.0000	0.9796	0.6042
				*p* (obs ≥ expected)	1.0000	1.0000	0.0600	0.7836
2008	LMN	Bulrush	4	No. Taxa	7	—	12	—
				C‐score	0.4286		0.8485	
				*p* (obs ≤ expected)	1.0000		0.7884	
				*p* (obs ≥ expected)	1.0000		0.4190	
2010	LHW	Bulrush	5	No. Taxa	10	8	17	7
				C‐score	0.4444	1.4643	0.6985	0.7619
				*p* (obs ≤ expected)	0.9870	0.9310	0.0288	0.8826
				*p* (obs ≥ expected)	0.1458	0.1024	0.9950	0.3646
2010	LMN	Bulrush	6	No. Taxa	16	6	28	14
				C‐score	1.3667	1.9333	0.9550	1.0000
				*p* (obs ≤ expected)	0.9080	0.5012	0.8110	0.7288
				*p* (obs ≥ expected)	0.1174	0.7350	0.2160	0.3124
2010	LMS	Lily	4	No. Taxa	14	8	30	12
				C‐score	0.6484	0.6786	0.7655	0.3333
				*p* (obs ≤ expected)	0.2624	0.9366	0.8220	0.4360
				*p* (obs ≥ expected)	0.9368	0.6084	0.2010	0.7866
2011	LES	Lily	7	No. Taxa	10	—	7	—
				C‐score	1.4889		0.7619	
				*p* (obs ≤ expected)	0.6380		1.0000	
				*p* (obs ≥ expected)	0.4982		0.0570	
2011	LHNE	Bulrush	10	No. Taxa	34	15	46	14
				C‐score	1.3743	2.6857	2.4580	2.1648
				*p* (obs ≤ expected)	0.4622	0.6696	0.9162	0.2068
				*p* (obs ≥ expected)	0.5578	0.3724	0.0894	0.8450
2011	LHSW	Bulrush	9	No. Taxa	25	10	31	14
				C‐score	1.7633	1.2222	1.1914	1.2198
				*p* (obs ≤ expected)	0.6100	0.0726	0.4728	0.4138
				*p* (obs ≥ expected)	0.4168	0.9634	0.5420	0.6680
2011	LHW	Bulrush	24	No. Taxa	37	13	66	20
				C‐score	6.4955	7.3333	6.8289	7.0790
				*p* (obs ≤ expected)	0.6944	0.8774	0.8648	0.6036
				*p* (obs ≥ expected)	0.3116	0.1302	0.1376	0.4090
2011	LHW	Lily	4	No. Taxa	**10**	7	20	8
				C‐score	**0.8222**	1.1429	0.7579	0.4286
				*p* (obs ≤ expected)	**0.9988**	0.7106	0.7580	0.8718
				*p* (obs ≥ expected)	**0.0054**	0.5232	0.2912	0.5956
2011	LMN	Bulrush	12	No. Taxa	34	11	49	12
				C‐score	1.3993	1.7636	2.2262	2.0606
				*p* (obs ≤ expected)	0.2090	0.7834	0.1554	0.7818
				*p* (obs ≥ expected)	0.8042	0.2548	0.8512	0.2486
2011	LON	Lily	8	No. Taxa	16	6	23	7
				C‐score	1.3917	1.0000	1.9289	0.5714
				*p* (obs ≤ expected)	0.4376	0.6380	0.6656	0.3262
				*p* (obs ≥ expected)	0.6276	0.6362	0.3660	0.8862
2011	LOS	Lily	4	No. Taxa	10	—	15	5
				C‐score	0.6444		0.6381	0.3000
				*p* (obs ≤ expected)	0.4258		0.9396	0.7538
				*p* (obs ≥ expected)	0.8226		0.0862	1.0000
2011	LS	Bulrush	5	No. Taxa	10	10	**27**	10
				C‐score	0.8222	1.1212	**0.7066**	0.6444
				*p* (obs ≤ expected)	0.8790	0.6304	**0.9996**	0.8492
				*p* (obs ≥ expected)	0.3350	0.4948	**0.0004**	0.3008
2011	LS	Lily	5	No. Taxa	9	—	20	14
				C‐score	0.8889		0.5684	0.8462
				*p* (obs ≤ expected)	0.7800		0.0482	0.4152
				*p* (obs ≥ expected)	0.3756		1.0000	0.6858
2012	LES	Lily	5	No. Taxa	14	—	16	6
				C‐score	0.6264		1.0500	0.3333
				*p* (obs ≤ expected)	0.6394		0.4890	0.4748
				*p* (obs ≥ expected)	0.5458		0.6264	1.0000
2012	LHNE	Bulrush	5	No. Taxa	16	8	**23**	11
				C‐score	0.5167	0.8571	**0.9130**	1.2182
				*p* (obs ≤ expected)	0.1382	0.5540	**0.9966**	0.7482
				*p* (obs ≥ expected)	0.9764	0.7586	**0.0046**	0.3742
2012	LHNE	Lily	6	No. Taxa	14	6	28	11
				C‐score	1.1429	1.1333	1.3571	0.8364
				*p* (obs ≤ expected)	0.4498	0.7694	0.6398	0.1362
				*p* (obs ≥ expected)	0.6782	0.4462	0.4006	0.9406
2012	LHSW	Bulrush	6	No. Taxa	17	9	28	11
				C‐score	1.0294	1.0278	1.2566	0.8546
				*p* (obs ≤ expected)	0.5572	0.6366	0.8654	0.5792
				*p* (obs ≥ expected)	0.5070	0.5266	0.1614	0.5944
2012	LHW	Bulrush	**7**	No. Taxa	**25**	11	40	13
				C‐score	**1.2567**	1.0909	1.3410	1.3077
				*p* (obs ≤ expected)	**0.9954**	0.3358	0.9560	0.6604
				*p* (obs ≥ expected)	**0.0050**	0.7234	0.0464	0.4050
2012	LMN	Bulrush	8	No. Taxa	22	12	33	13
				C‐score	1.7273	1.2879	1.6345	1.0256
				*p* (obs ≤ expected)	0.6830	0.1228	0.9016	0.5670
				*p* (obs ≥ expected)	0.3452	0.9362	0.1064	0.5112
2012	LMN	Lily	5	No. Taxa	13	7	22	11
				C‐score	0.7308	0.6191	0.8615	0.5091
				*p* (obs ≤ expected)	0.6260	0.3088	0.9496	0.2320
				*p* (obs ≥ expected)	0.5338	1.0000	0.0616	1.0000
2012	LON	Lily	6	No. Taxa	12	8	30	10
				C‐score	1.4242	1.0357	1.1448	0.6444
				*p* (obs ≤ expected)	0.7536	0.7752	0.5224	0.5148
				*p* (obs ≥ expected)	0.3300	0.5558	0.5200	0.6962
2013	LES	Lily	5	No. Taxa	10	5	20	8
				C‐score	0.4000	1.0000	0.5053	1.1786
				*p* (obs ≤ expected)	0.9154	0.5768	0.9530	0.8534
				*p* (obs ≥ expected)	1.0000	0.9206	0.0656	0.3618
2013	LHSW	Bulrush	4	No. Taxa	14	9	24	8
				C‐score	0.7143	0.6389	0.7355	0.4286
				*p* (obs ≤ expected)	0.6322	0.9464	0.2960	0.4752
				*p* (obs ≥ expected)	0.5454	0.2336	0.8110	0.9212
2013	LHW	Bulrush	5	No. Taxa	21	**12**	34	10
				C‐score	0.7238	**1.1212**	1.0998	0.6000
				*p* (obs ≤ expected)	0.6116	**0.9998**	0.5704	0.8612
				*p* (obs ≥ expected)	0.4780	**0.0006**	0.4658	0.4934

*Note:* Eco‐region abbreviations are as follows: LES, Lake Erie South; LHNE, Lake Huron Northeast; LHSW, Lake Huron Southwest; LHW, Lake Huron West; LMN, Lake Michigan North; LMS, Lake Michigan South; LON, Lake Ontario North; LOS, Lake Ontario South; LS, Lake Superior. Results that were significantly different (*α* = 0.05) from random distribution are represented in bold. Community matrices that did not meet the minimum matrix size for analysis are designated with “—”.

**TABLE 4:** Null model analysis outcomes based upon the C‐score index for macroinvertebrate communities categorized by taxonomic classification (Family) including Baetidae, Chironomidae, Coenagrionidae, Corixidae, Dytiscidae, and Gerridae.

Year	Ecoregion	Vegetation	Sites		BAE	CHI	COE	COR	DYT	GER
2000	LMN	Lily	6	No. Taxa	—	—	—	5	—	—
				C‐score				0.2000		
				*p* (obs ≤ expected)				1.0000		
				*p* (obs ≥ expected)				1.0000		
2002	LHSW	Bulrush	6	No. Taxa	—	5	—	—	—	—
				C‐score		0.1000				
				*p* (obs ≤ expected)		1.0000				
				*p* (obs ≥ expected)		1.0000				
2002	LHW	Bulrush	11	No. Taxa	—	5	—	—	7	—
				C‐score		2.0000			0.9524	
				*p* (obs ≤ expected)		0.6238			0.9816	
				*p* (obs ≥ expected)		1.0000			0.4078	
2002	LMN	Bulrush	10	No. Taxa	—	5	—	—	8	—
				C‐score		0.6000			1.7143	
				*p* (obs ≤ expected)		1.0000			0.6532	
				*p* (obs ≥ expected)		0.1940			0.5034	
2002	LMN	Lily	6	No. Taxa	—	—	—	—	5	—
				C‐score					0.5000	
				*p* (obs ≤ expected)					1.0000	
				*p* (obs ≥ expected)					1.0000	
2004	LHSW	Bulrush	4	No. Taxa	—	5	—	—	—	—
				C‐score		0.1000				
				*p* (obs ≤ expected)		1.0000				
				*p* (obs ≥ expected)		1.0000				
2010	LMN	Bulrush	6	No. Taxa	—	5	—	—	—	—
				C‐score		0.3000				
				*p* (obs ≤ expected)		0.7404				
				*p* (obs ≥ expected)		1.0000				
2011	LES	Lily	7	No. Taxa	—	5	—	—	—	—
				C‐score		0.9000				
				*p* (obs ≤ expected)		0.6646				
				*p* (obs ≥ expected)		1.0000				
2011	LHNE	Bulrush	10	No. Taxa	—	6	—	5	6	—
				C‐score		0.0667		2.2000	0.7333	
				*p* (obs ≤ expected)		1.0000		0.6852	0.6834	
				*p* (obs ≥ expected)		1.0000		0.6916	1.0000	
2011	LHSW	Bulrush	9	No. Taxa	—	5	—	—	—	—
				C‐score		3.6000				
				*p* (obs ≤ expected)		0.9742				
				*p* (obs ≥ expected)		0.0360				
2011	LHW	Bulrush	24	No. Taxa	6	**8**	5	—	9	5
				C‐score	12.8000	**6.7857**	5.3000		2.7778	3.9000
				*p* (obs ≤ expected)	0.8682	**0.9988**	0.3110		0.2714	0.6724
				*p* (obs ≥ expected)	0.1646	**0.0014**	1.0000		0.8280	0.4506
2011	LMN	Bulrush	12	No. Taxa	5	—	—	5	—	—
				C‐score	3.3000			2.4000		
				*p* (obs ≤ expected)	0.9546			0.7190		
				*p* (obs ≥ expected)	0.2530			0.4696		
2011	LON	Lily	8	No. Taxa	—	5	—	—	—	—
				C‐score		0.7000				
				*p* (obs ≤ expected)		0.5096				
				*p* (obs ≥ expected)		1.0000				
2011	LS	Bulrush	5	No. Taxa	—	5	—	—	—	—
				C‐score		1.2000				
				*p* (obs ≤ expected)		0.9120				
				*p* (obs ≥ expected)		0.2242				
2011	LS	Lily	5	No. Taxa	—	5	—	—	—	—
				C‐score		0.9000				
				*p* (obs ≤ expected)		1.0000				
				*p* (obs ≥ expected)		0.2326				
2012	LHSW	Bulrush	6	No. Taxa	—	—	—	—	5	—
				C‐score					0.8000	
				*p* (obs ≤ expected)					0.6476	
				*p* (obs ≥ expected)					1.0000	
2012	LON	Lily	6	No. Taxa	—	5	—	—	—	—
				C‐score		1.0000				
				*p* (obs ≤ expected)		0.4018				
				*p* (obs ≥ expected)		1.0000				

*Note:* Eco‐region abbreviations are as follows: LES, Lake Erie South; LHNE, Lake Huron Northeast; LHSW, Lake Huron Southwest; LHW, Lake Huron West; LMN, Lake Michigan North; LON, Lake Ontario North; LS, Lake Superior. Results that were significantly different (*α* = 0.05) from random distribution are represented in bold. Community matrices that did not meet the minimum matrix size for analysis are designated with “—”.

**TABLE 5:** Null model analysis outcomes based upon the C‐score index for macroinvertebrate communities categorized by taxonomic group (Family) including Hydrophilidae, Leptoceridae, Libellulidae, Lymnaeidae, and Planorbidae.

Year	Ecoregion	Vegetation	Sites		HYD	LEP	LIB	LYM	PLN
2002	LHW	Bulrush	11	No. Taxa	—	5	—	5	—
				C‐score		2.1000		2.1000	
				*p* (obs ≤ expected)		0.3634		0.6484	
				*p* (obs ≥ expected)		0.9158		0.7996	
2002	LMN	Bulrush	10	No. Taxa	—	5	—	—	—
				C‐score		4.3000			
				*p* (obs ≤ expected)		0.7980			
				*p* (obs ≥ expected)		0.3210			
2002	LMN	Lily	6	No. Taxa	—	—	—	—	5
				C‐score					0.4000
				*p* (obs ≤ expected)					0.4566
				*p* (obs ≥ expected)					1.0000
2011	LHSW	Bulrush	9	No. Taxa	—	6	—	—	—
				C‐score		1.6667			
				*p* (obs ≤ expected)		0.5580			
				*p* (obs ≥ expected)		0.6094			
2011	LHW	Bulrush	24	No. Taxa	5	—	—	—	**5**
				C‐score	2.1000				**10.3000**
				*p* (obs ≤ expected)	0.7016				**0.9978**
				*p* (obs ≥ expected)	0.4136				**0.0038**
2011	LMN	Bulrush	12	No. Taxa	—	—	—	—	5
				C‐score					1.3000
				*p* (obs ≤ expected)					0.3894
				*p* (obs ≥ expected)					0.9114
2012	LMN	Lily	5	No. Taxa	—	5	—	—	—
				C‐score		0.5000			
				*p* (obs ≤ expected)		1.0000			
				*p* (obs ≥ expected)		1.0000			
2012	LON	Lily	6	No. Taxa	—	—	5	—	—
				C‐score			1.1000		
				*p* (obs ≤ expected)			0.6700		
				*p* (obs ≥ expected)			0.6822		

*Note:* Eco‐region abbreviations are as follows: LES, Lake Erie South; LHNE, Lake Huron Northeast; LHSW, Lake Huron Southwest; LHW, Lake Huron West; LMN, Lake Michigan North; LON, Lake Ontario North; LS, Lake Superior. Results that were significantly different (*α* = 0.05) from random distribution are represented in bold. Community matrices that did not meet the minimum matrix size for analysis are designated with “—”.

SES distribution was significantly different (greater than zero) for community matrices classified by trophic group, but not by taxonomic group (Figure 3). When communities are classified based on trophic classification (i.e., functional feeding group) significantly more assembly structure than expected by chance is observed. Although only 6.7% of community matrices categorized by trophic group exhibited non‐random co‐occurrence structure, observations align with the distribution of SES.

**FIGURE 4:**
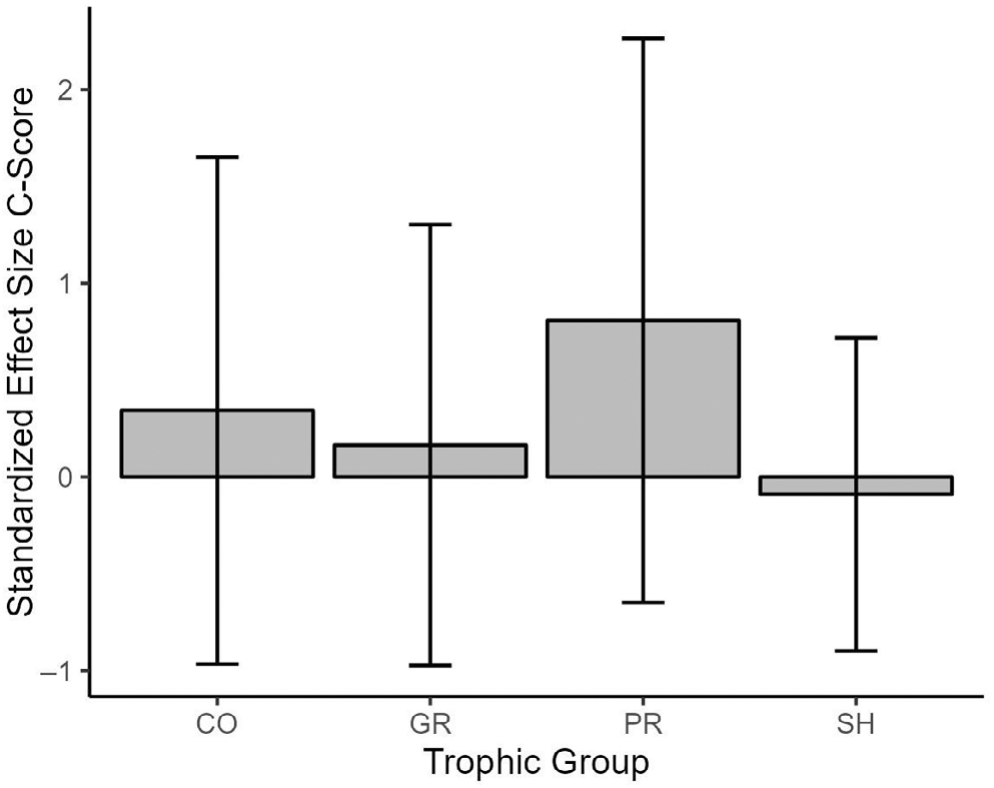
The influence of trophic group (i.e., functional feeding group) on C‐Score Standardized Effect Size (SES). The Predator (PR) functional feeding group had a significantly higher mean SES C‐Score than both the Grazer (GR) and Shredder (SH) functional feeding group mean SES C‐Score. However, no statistically significant difference was seen between PR and collector (CO) functional feeding groups. All means fell within ±2 SD given a 95% confidence interval.

**FIGURE 5:**
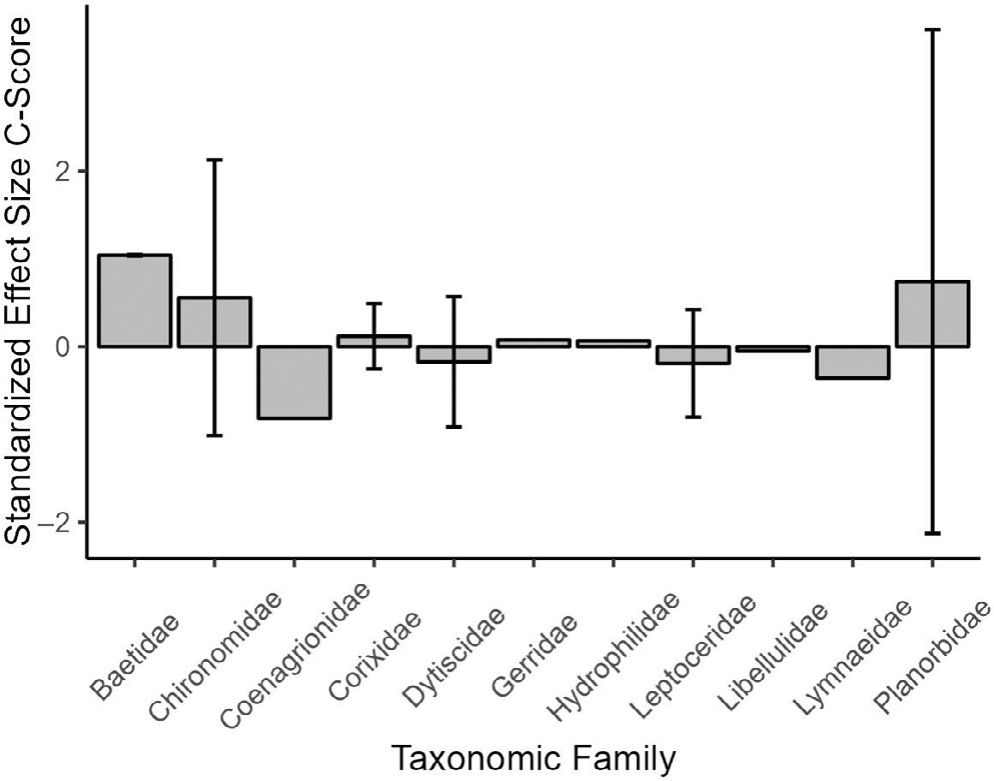
The influence of taxonomic classification (Family) on standardized effect size. Mean SES CScores were not significantly different from each other among taxonomic classifications.

Furthermore, functional feeding group designation had a significant effect on SES within the functional feeding group matrices, whereby SES for the Predator functional feeding group were higher than all other functional feeding group designations and significantly higher than Grazer (*p* = 0.0279) and Shredder (*p =* 0.0032) SES (Figure 4). A similar pattern was not observed based on family groups within community matrices classified based on the taxonomic group (Figure 5).

### 3.2 Investigating Instances of Non‐random Community Structure

Our study design (Figure 1) provides a framework to isolate alternative hypotheses for mechanisms leading to non‐random co‐occurrence patterns. We only observed negative co‐occurrences, so the first step was to test for differential pollution tolerance. Among the nine non‐random associations, we did not discern any clear separation of pollution tolerance among the interacting taxa (see Data S1). The next step was to assess differential microhabitat affinities (having controlled for macrohabitats in the study design). Microhabitat data were lacking for three of the non‐random communities. Among the six remaining, we found that the environmental characteristics of sites differed significantly among sites for two of the communities. In these two cases, parsimony suggests that microhabitat affinities may contribute to the non‐random assembly structure (i.e., there is no conclusive support for competition as a driver). Having eliminated other alternative hypotheses, we can conclude that the non‐random negative co‐occurrence patterns we observed in the remaining four cases could be the result of competitive exclusion. It should be noted that in all nine non‐random co‐occurrence cases, the taxa involved were known to overwinter in wetlands (see Data S1).

## 4 Discussion

Non‐random community structure was rare albeit not nonexistent for Great Lake coastal wetland macroinvertebrates and could be due to the high turnover related to intense disturbance regimes (Kagalou et al. 2006; Langer et al. 2016; Stewart and Schriever 2022). The few instances of non‐random co‐occurrence (6.7% or 9 of 134 individual cases) were all negative and interestingly contained taxa with the relatively rare ability to overwinter in the wetlands. Although each community matrix is unique and independent relative to the others, each is susceptible to type I and II errors. Our matrix sizes were small to intermediate relative to past power analysis studies, which show low error rates (Fayle and Manica 2010; Gotelli and Ulrich 2012). Given the total number of matrices analyzed, the proportion we assessed as non‐random is within the realm of possible through type I error; however, analysis of SES for each biological categorization supports our observed proportion of non‐random outcomes. Past studies of other taxa have demonstrated non‐random community structure in larger proportions of matrices (e.g., 56% of 124 matrices, Horner‐Devine et al. 2007 and 42% of 269 matrices Pitta et al. 2012). In contrast, Sfenthourakis et al. (2006) found little evidence of pairwise species interactions (among congeners or more broadly) among terrestrial isopods, which is consistent with our findings.

Regardless of the risk of type I errors, the nine instances of nonrandom community structure were interesting. We were able to systematically eliminate alternative mechanisms to conclude in at least four of the cases competition was the most likely mechanism. We were able to eliminate pollution as a driver in all nine cases but only had environmental data for six of the nine site × taxa matrices to test for shared/differential habitat affinities. In two of those six cases, we did observe microhabitat differences among non‐randomly distributed taxa, thus not allowing us to rule out either microhabitat affinities or competition. But, in the remaining four cases, pollution tolerance and microhabitats were highly comparable across involved taxa and sites suggesting that competition may have been the dominant driver of the negative co‐occurrences in these cases. What makes these rare instances interesting is, as mentioned, the core taxa exhibiting most of the negative co‐occurrences are all known to overwinter in wetlands, making them permanent residents without the need for recolonization and likely increasing competition relative to species engaged in a more lottery recolonization strategy (Sale 1978). This suggests that as winters become less severe conditions are likely to become more hospitable, reducing the presently high turnover and beta‐level diversity (Langer et al. 2016, Stewart and Schriever 2022). This may allow the community to reach levels approaching carrying capacity (reduced disturbance impacts) leading to an increase in competitive interactions and subsequent changes to community structure and biodiversity patterns.

Increasing environmental variability, as well as dispersal limitation, have been shown to increase the effects of turnover on macroinvertebrate communities over time (Angeler 2013; Stewart and Schriever 2022). Additionally, extreme, episodic disturbance events followed by colonization have been shown to result in unique communities even though all other conditions remain similar to prior observations (Shea and Chesson 2002). Coastal wetlands not only experience a multitude of natural disturbances (e.g., wave action, extreme fluctuation of dissolved oxygen levels, and ice scour) but are characterized by them (Burton, Uzarski, and Genet 2004; Cooper, Lamberti, and Uzarski 2014; Minc and Albert 1998). Extreme physical and chemical changes during winter represent inhospitable conditions for macroinvertebrates in Great Lakes coastal wetlands; however, some taxa can tolerate freeze or find refuges (e.g., senescence in the substrate; Danks 2007; Partridge 2001). Those macroinvertebrates unable to overwinter on‐site must immigrate back into wetlands via passive or active dispersal upon ice melt and re‐establishment of vegetation. In the spring, coastal wetlands experience an influx of taxa via re‐emergence, immigration via wind or current dispersal, and egg deposition. It seems this disturbance regime and recolonization are the likely dominant drivers of the observed patterns of random co‐occurrence, but less severe winters projected in the future (Wuebbles and Hayhoe 2004) may alter these community processes.

Our study design effectively removed competing hypotheses (Blanchet, Cazelles, and Gravel 2020) and isolated competitive mechanisms (Figure 1). The minority of communities, those that exhibited non‐random structure, included predominately taxa capable of overwintering within wetland habitats frozen in ice or sediments including two snail species, *Gyraulus* (Planorbidae) and *Planorbis* (Planorbidae) (Herrmann and Harman 1975; Olsson 1981, 1984, 1988; Smith 1991a), and multiple taxa of non‐biting midges (Chironomidae.) Additionally, odonate nymphs (e.g., *Enallagma* and *Coenagrion*) are documented to overwinter frozen in ice (Daborn 1971; Moore and Lee Jr 1991). Odonates dominated the Predator functional feeding groups among which we saw multiple instances of non‐random (negative) co‐occurrence.

The characteristic zonation of vegetation in Great Lakes coastal wetlands is a function of wave action, which subsequently affects organic sediment depth among other structural characteristics of a site (Burton, Uzarski, and Genet 2004). Vegetation, substrate, other physical and chemical attributes, and available niche spaces that provide for competitive interactions may be dictated more by the intensity of disturbance experienced at the community level (Menge and Sutherland 1987). We suggest that sites exhibiting non‐random co‐occurrence experience less disturbance, facilitating overwinter survival, and giving those taxa a competitive advantage over recolonizing taxa.

We suggest that harsh winter conditions serve as an annual disturbance that maintains communities below carrying capacity and facilitates predominately random co‐occurrence patterns across Great Lake coastal wetlands. This hypothesis has interesting ramifications and points to the need for further research into winter severity and the effects of climate change on community assembly. Winter severity, and specifically ice cover, is predicted to decrease in the future (Hayhoe et al. 2010; Smith 1991b; Wuebbles and Hayhoe 2004). We can then predict higher overwintering success of a broader suite of taxa leading to wetland communities more likely to approach carrying capacity leading to increased competitive interactions and a greater probability of competitive exclusion. Changes in disturbance regime, structuring forces (i.e., decreased importance on the colonization process and an increase in competitive interactions) may result in changes to biodiversity (e.g., redundancy) and ultimately affect ecological functioning. These predictions could be tested by exploring biodiversity and co‐occurrence structure in wetlands facing less severe disturbance regimes and certainly may have profound implications for the ecological functioning of wetlands under future climate scenarios.

## Author Contributions

**Alexandra A. Bozimowski:** conceptualization (lead), data curation (lead), formal analysis (lead), investigation (lead), methodology (lead), visualization (lead), writing – original draft (lead). **Brent A. Murry:** conceptualization (supporting), formal analysis (supporting), methodology (equal), visualization (supporting), writing – original draft (equal). **Donald G. Uzarski:** conceptualization (supporting), funding acquisition (lead), writing – review and editing (supporting).

## Conflicts of Interest

The authors declare no conflicts of interest.

### Open Research Badges

This article has earned an Open Data badge for making publicly available the digitally‐shareable data necessary to reproduce the reported results. The data is available at https://doi.org/10.5061/dryad.3tx95x6nw.

## Supporting information


Data S1.

## Data Availability

Presence–absence matrix data, standardized effect size (SES) values, and associated metadata are available at https://doi.org/10.5061/dryad.3tx95x6nw. Additional information about sample collection and the Great Lakes Coastal Wetland Monitoring Program can be found at greatlakeswetlands.org.
