# Supplementary material for: Co‐Occurrence Patterns of Aquatic Macroinvertebrates in Laurentian Great Lakes Coastal Wetlands
Source: Ecol Evol. 2024 Dec 2;14(12):e70622. doi: 10.1002/ece3.70622 (PMC11612260; doi:10.1002/ece3.70622)
Supplement: Supplementary file 1 — Data S1. [file ECE3-14-e70622-s001.pdf]

Northern Lake Michigan, 2000, Collector FFG								
		Sites						
		**1301**	**1297**	**1281**	**1305**	**LR**		**Tolerance**
**Taxa**	**Ancyronyx spp.**	0	0	1	0	0		6
	**Brachycercus spp.**	0	0	1	0	0		3
	**Caenis spp.**	0	1	0	1	0		7
	**Callibaetis spp.**	1	1	0	1	0		9
	**Centroptilum spp.**	0	0	1	0	0		2
	**Chironomini spp.**	1	1	1	1	1		8
	**Dpolymorpha spp.**	0	0	1	0	0		UNK
	**Dubiraphia spp.**	0	0	1	0	0		8
	**Hexagenia spp.**	1	0	1	1	0		6
	**Orthocladiinae spp.**	1	1	0	1	1		6
	**Tanytarsini spp.**	1	0	0	0	0		6
	**Tropisternus spp.**	0	0	1	1	1		10

Northern Lake Michigan, 2002, Shredder FFG													
		Sites											
		1301	LR	1598	1487	1512	1494	1487	1516	1515	1519		Tolerance
Taxa	Rhinoncus spp.	1	0	0	0	0	0	0	0	0	0		UNK
	Haliplus spp.	0	0	0	0	1	1	0	0	0	0		8
	Peltodytes spp.	0	0	0	0	0	1	0	0	0	1		4
	Acentria spp.	1	0	0	1	0	0	0	0	1	0		1
	Nectopsyche spp.	1	0	1	1	1	0	1	1	1	0		3
	Triaenodes spp.	0	0	0	0	0	1	0	0	0	0		6
	Limnephilus spp.	0	0	1	0	0	0	0	1	0	1		3
	Gyraulus spp.	1	1	1	1	1	1	1	0	1	1		5.5
	Planorbella spp.	0	0	0	1	1	1	0	0	1	1		UNK
	Promenetus spp.	1	0	0	0	0	0	0	0	0	0		UNK
	Crangonyx spp.	0	0	0	0	0	1	0	0	0	1		8
	Gammarus spp.	1	1	1	1	1	1	1	1	1	1		4
	Hyallela azteca	1	1	1	1	1	1	1	1	1	1		8
	Caecidotea spp.	1	1	0	0	1	1	0	0	0	1		8
	Lirceus spp.	0	0	0	0	0	1	0	0	1	1		2

Western Lake Huron, 2002, Predator FFG

		Sites											
		538	615	617	613	635	630	637	642	704	499		Tolerance
Taxa	Acilius spp.	1	0	0	0	0	0	0	0	0	0		UNK
	Coptotomus spp.	1	0	0	0	0	0	0	0	0	0		UNK
	Hydroporus spp.	1	0	0	0	0	1	0	0	0	0		5
	Ilybius spp.	1	0	0	0	0	0	0	0	0	0		6
	Laccophilus spp.	0	0	1	0	0	0	0	0	0	0		6
	Liodessus spp.	0	0	0	0	0	0	0	0	1	1		7
	Rhantus spp.	1	0	0	0	0	0	0	0	0	0		UNK
	Dineutus spp.	0	1	0	0	0	0	0	0	0	0		3.7
	Gyrinus spp.	1	0	0	0	0	0	1	0	0	0		9
	Bezzia spp.	1	1	1	1	0	1	1	0	1	1		6
	Culicoides spp.	0	0	1	0	0	0	0	0	0	0		10
	Probezzia spp.	0	0	0	0	1	0	0	1	0	1		6
	Tanypodinae spp.	0	1	1	1	1	0	1	1	1	1		8
	Belostoma spp.	1	0	1	1	0	1	1	1	1	1		UNK
	Palmacorixa spp.	0	0	0	1	1	1	1	1	0	0		2
	Sigara spp.	0	1	1	1	0	0	1	1	0	0		4
	Trichocorixa spp.	0	1	1	1	1	0	0	1	1	0		5
	Gerris spp.	0	0	0	0	0	0	0	0	1	0		UNK
	Trepobates spp.	1	0	0	0	0	1	0	0	0	0		5
	Hydrometra spp.	0	0	0	0	0	1	0	0	0	0		UNK
	Mesovelia spp.	0	1	1	1	1	1	1	1	1	1		6
	Ranatra spp.	0	0	1	0	0	0	1	0	1	0		4
	Buenoa spp.	1	0	0	0	0	0	0	0	0	0		UNK
	Notonecta spp.	1	0	1	1	0	0	1	1	0	0		3
	Neoplea spp.	0	0	0	0	0	0	0	0	0	1		3
	Microvelia spp.	1	0	0	0	0	0	0	1	0	0		1
	Paravelia spp.	1	0	0	0	0	0	0	0	0	0		UNK
	Aeshna spp.	0	0	0	0	0	1	0	0	1	0		5
	Anax spp.	1	1	1	1	1	1	1	1	1	0		8
	Basiaeschna spp.	0	0	0	1	0	0	0	0	0	0		6
	Enallagma spp.	0	0	1	0	1	0	1	0	0	1		8
	Ischnura spp.	0	0	1	1	1	0	1	0	0	1		9
	Epitheca spp.	0	0	1	1	0	0	0	0	0	0		7
	Lestes spp.	1	0	0	0	0	0	0	0	0	0		9
	Libellula spp.	0	0	1	0	1	0	1	0	1	0		9
	Sympetrum spp.	1	1	0	0	1	1	0	0	1	0		10
	Oecetis spp.	0	0	0	0	0	0	0	0	1	1		8

Southern Lake Michigan, 2004, Collector FFG							
		Sites					
		Site 1	Site 2	Site 3	Site 4		Tolerance
Taxa	**Crenitis spp.**	0	0	1	0		UNK
	**Derallus spp.**	0	0	0	1		UNK
	**Tropisternus spp.**	0	0	1	0		10
	**Chironominae spp.**	1	1	0	1		8
	**Orthocladiinae spp.**	1	1	0	1		6
	**Tanytarsini spp.**	1	1	0	1		6
	**Callibaetis spp.**	1	1	0	0		9
	**Caenis spp.**	1	0	0	1		7
	**Hexagenia spp.**	1	1	0	0		6
	**Dreissena spp.**	0	0	0	1		UNK

Western Lake Huron, 2011, Collector FFG							
		Sites					
		613	660	816	917		Tolerance
Taxa	Chironomini spp.	1	1	0	1		8
	Orthocladiinae spp.	1	1	0	1		6
	Tanytarsini spp.	1	1	0	1		6
	Callibaetis spp.	1	0	0	1		9
	Centroptilum spp.	0	0	1	0		2
	Cloeon spp.	0	1	0	0		4
	Caenis spp.	1	1	1	1		7
	Eurylophella spp.	0	0	1	0		5
	Hexagenia spp.	1	1	0	1		6
	Pisidium spp.	1	1	0	0		7

Lake Superior, 2011, Predator FFG								
		Sites						
		969	976	979	1077	5210		Tolerance
Taxa	Helobdella spp.	1	0	0	0	0		7
	Agabus spp.	1	0	0	0	0		6
	Celina spp.	1	0	0	0	0		5
	Hydroporus spp.	1	0	0	0	0		5
	Laccophilus spp.	1	1	0	0	0		6
	Dineutus spp.	0	1	0	0	0		3.7
	Gyrinus spp.	1	1	0	0	0		9
	Bezzia spp.	1	0	0	0	1		6
	Tanypodinae spp.	1	0	0	0	0		8
	Belostoma spp.	1	0	0	0	0		UNK
	Palmacorixa spp.	0	1	1	1	0		2
	Trichocorixa spp.	1	1	0	0	0		5
	Aquarius spp.	1	0	0	0	0		5
	Trepobates spp.	0	0	0	0	1		5
	Merragata spp.	1	0	0	0	0		UNK
	Hydrometra spp.	1	0	0	0	0		UNK
	Mesovelia spp.	1	0	0	0	1		6
	Ranatra spp.	1	0	0	0	0		4
	Notonecta spp.	0	1	0	0	1		3
	Neoplea spp.	1	0	0	0	0		3
	Aeshna spp.	1	0	0	0	1		5
	Enallagma spp.	0	1	1	0	1		8
	Epitheca spp.	1	0	0	0	0		7
	Leucorrhinia spp.	1	0	0	0	1		UNK
	Libellula spp.	1	0	0	0	1		9
	Sympetrum spp.	1	0	0	0	0		10
	Oecetis spp.	1	1	0	1	0		8

Western Lake Huron, 2012, Collector FFG										
		**Sites**								
		**496**	**629**	**776**	**636**	**721**	**780**	**617**		**Tolerance**
**Taxa**	**Berosus spp.**	1	0	0	0	0	0	0		8
	**Tropisternus spp.**	1	0	1	0	1	0	0		10
	**Dasyhelea spp.**	0	0	0	0	0	0	1		5
	**Chironomini spp.**	1	1	1	1	1	1	1		8
	**Corynoneura spp.**	0	0	0	0	0	0	1		7
	**Orthocladiinae spp.**	1	1	1	1	1	1	1		6
	**Tanytarsini spp.**	1	1	1	1	1	1	1		6
	**Chrysops spp.**	0	0	0	0	0	1	0		6
	**Baetis spp.**	0	0	1	0	0	0	0		4
	**Callibaetis spp.**	0	0	1	0	0	0	1		9
	**Centroptilum spp.**	0	0	0	0	1	0	1		2
	**Cloeon spp.**	0	1	1	0	0	1	0		4
	**Brachycercus spp.**	1	0	0	0	0	0	0		3
	**Caenis spp.**	1	1	1	1	1	1	1		7
	**Attenella spp.**	0	0	0	0	0	0	1		3
	**Eurylophella spp.**	0	1	1	0	0	1	1		5
	**Ephemera spp.**	0	0	0	0	0	0	1		1
	**Hexagenia spp.**	0	1	0	0	0	1	0		6
	**Oxyethira spp.**	0	0	0	1	0	0	1		3
	**Mystacides spp.**	1	0	0	0	1	0	0		4
	**Ylodes spp.**	0	0	0	0	0	1	0		UNK
	**Cernotina spp.**	0	0	0	1	0	0	1		6
	**Dpolymorpha spp.**	0	0	0	0	0	0	1		UNK
	**Pisidium spp.**	1	0	0	1	1	0	0		7
	**Bithynia spp.**	1	0	0	1	0	0	0		UNK

Northeast Lake Huron, 2012, Predator FFG								
		Sites						
		5746	5791	5792	5900	6057		Tolerance
Taxa	Helobdella spp.	0	1	1	1	0		7
	Hygrotus spp.	0	0	0	1	0		4
	Laccophilus spp.	0	0	0	0	1		6
	Liodessus spp.	0	1	1	0	0		7
	Dineutus spp.	1	1	1	0	1		3.7
	Bezzia spp.	1	1	1	1	1		6
	Tanypodinae spp.	1	1	1	1	1		8
	Cenocorixa spp.	0	0	0	1	0		UNK
	Trichocorixa spp.	0	0	0	0	1		5
	Mesovelia spp.	0	0	0	1	0		6
	Ranatra spp.	0	1	0	0	0		4
	Notonecta spp.	0	0	0	1	0		3
	Neoplea spp.	0	0	0	1	0		3
	Sialis spp.	1	0	0	0	0		4
	Basiaeschna spp.	0	1	0	0	0		6
	Enallagma spp.	1	0	1	0	0		8
	Ischnura spp.	1	1	1	0	0		9
	Epitheca spp.	0	0	1	0	0		7
	Gomphus spp.	0	0	0	0	1		5
	Ladona spp.	0	0	0	1	0		UNK
	Leucorrhinia spp.	0	0	0	1	0		UNK
	Oecetis spp.	0	1	1	0	0		8
	Hydra spp.	0	0	0	1	0		9

Western Lake Huron, 2013, Grazer FFG								
		Sites						
		590	591	736	790	777		Tolerance
Taxa	Stenonema spp.	0	0	0	0	1		4
	Helicopsyche spp.	0	0	0	0	1		3
	Ferrissia spp.	0	0	0	0	1		8
	Laevapex spp.	0	0	1	0	0		UNK
	Acella spp.	0	0	0	0	1		UNK
	Fossaria spp.	1	1	1	1	0		2.6
	Pseudosuccinea columella	1	1	0	0	0		7
	Stagnicola spp.	1	0	0	1	0		7
	Physa spp.	1	1	1	1	1		5
	Valvata spp.	0	0	1	0	0		0
	Amnicola spp.	0	0	1	1	0		5
	Goniobasis spp.	0	0	0	0	1		UNK
